# A situation analysis of psychiatrists in South Africa’s rural primary healthcare settings

**DOI:** 10.4102/phcfm.v9i1.1335

**Published:** 2017-05-29

**Authors:** Johannes H. De Kock, Basil J. Pillay

**Affiliations:** 1 Department of Behavioural Medicine, University of KwaZulu-Natal, South Africa

## Abstract

**Background:**

South Africa (SA) has been facing serious challenges in providing human resources for the delivery of essential mental health (MH) services. The majority of its prescribing MH specialists, psychiatrists, practise in private, urban and peri-urban areas. The findings of a situation analysis audit of psychiatrist human resources in the public rural primary healthcare (PRPHC) sector are presented in this paper.

**Method:**

This audit was based on both primary and secondary data. The primary data were obtained from key informant interviews with the clinical heads of 160 PRPHC facilities, while the secondary data comprised a literature review.

**Results:**

The results indicate that psychiatrists are severely underrepresented, employed at a rate of 0.03 per 100 000 population in SA’s PRPHC settings.

**Conclusions:**

Because of a lack of MH nurses and medical officers dedicated to MH in PRPHC facilities, recommendations are made that the current task shifting strategy be revisited to include more cadres of MH professionals with specialised psychopharmacological training, as non-medical prescribers at PRPHC level. It is advised that visiting psychiatrists and family physicians be involved in the construction of training and supervision programmes for non-medical prescribers at the primary healthcare level.

## Introduction

By 1994, South Africa’s (SA) psychiatric services were confined to a fragmented hospi-centric approach, disjointed along racial lines and limited to metropolitan areas.^1^ SA’s psychiatry training programmes had historically been deemed to be at a high standard, but only a small, exclusive portion of its population – those able to afford private medical aid – had access to this care. It is estimated that more than 30 million South Africans were dependent on the public health system by 1994,^2^ a sector in which less than 170 psychiatrists were practising at the time. Of these psychiatrists, less than 5% worked in rural areas, which included more than 40% of SA’s population.^3,4^

At the advent of SA’s democracy, the psychiatric community was outspoken about increasing accessibility to psychiatric services, especially to the underserved rural areas that are served mainly by the country’s primary healthcare (PHC) system.^1,3,5^ The profession of psychiatry has been at the forefront of lobbying to increase inclusivity of mental healthcare (MHC) for South Africans. It was the Society of Psychiatrists of SA (SPSA) that contributed greatly to the development of a framework from which the National Department of Health (NDOH) could include mental health (MH) in the country’s PHC system. Shortly after the advent of democracy in SA, the NDOH had adopted the approach of decentralising MHC towards a community-based service, integrated into PHC.^5^ The integration of MH into the wider context of PHC was (and is) a difficult task because of budgetary restrictions with MHC receiving a disproportionately small portion of the country’s health budget,^6^ limited MH human resources (with inadequate MH training) and high patient loads in these settings.^7^ The SA Society of Psychiatrists (SASOP) replaced the SPSA in 2006 and has been even more active in efforts to address the gap between the mental illness burden of disease and treatment options available.^8,9,10^ Despite their best efforts, SA, a high-middle–income country (HMIC) has continued to face serious challenges in providing human resources for the delivery of essential MH services with workforce numbers well below of what could be expected even from a middle income country.^7,11^

The vast majority of its prescribing MH specialists – psychiatrists – continue to practise in urban areas.^6^ The World Health Organization (WHO) advocates a task shifting approach to alleviate medical workforce shortages.^12^ Task shifting suggests that clinical tasks, including prescribing certain medications, are shifted to cadres of non-medical health professionals with specialised training in a specific field.^13^ While recent studies suggest that it is in the public rural primary healthcare (PRPHC) sector where access to psychiatrists remain the lowest,^14^ there remains a knowledge gap with regard to this cadre’s human resources and services in these areas. This audit, as a part of a larger situation analysis with the objective of ascertaining the medical and non-medical MH workforce at the PRPHC level,^15^ aimed primarily to fill the knowledge gap pertaining to psychiatrists and medical officers (MOs) dedicated to MH care. A secondary aim was to review strategic opportunities to expand the current task shifting approach in an attempt to improve access to care in these areas.

## Methods

### Participants

The clinical heads of 160 (98%) of SA’s 163 PRPHC health facilities participated in the situation analysis.

### Procedure

#### Secondary data

The data sources for this audit included both primary and secondary data. Our secondary data analysis was done in the form of a desk review that was based on validated search strategies^16^ to obtain literature on human resources for MH in low- and middle income countries (LAMICS). Domestic sources comprised government departments’ and governing bodies’ reports including the NDOH, the SPSA and SASOP, the Health Professions Council of SA (HPCSA), the Health Systems Trust and the Colleges of Medicine of SA. The census data of 2011 and national household survey data from StatsSA were also included^17^ to ascertain the population’s use of health facilities. International data were collected from the WHO’s reports on global MH human resources and services.^11,18,19^ Local and global scholarly works on MH human resources and services were accessed by making use of the electronic academic search engines.

#### Primary data

The secondary data gathering and perusal preceded and informed the primary data gathering and indicated that eight out of the nine SA provinces had health facilities classified as rural (Gauteng being the only province that does not have rural public health facilities).^20^ The eight participating provinces established that the Department of Health’s (DOH’s) Human Resource Management Circular of 2004^20^ was used to classify their health facilities as rural. In partnership with the participating provinces’ DOHs, a list consisting of 163 rural health facilities was identified to be included as PRPHC facilities in this audit. PRPHC facilities are classified as rural, difficult to access health services centres (district hospitals, primary care clinics and community health centres) in the public sector.^20^ The contact details of these 163 health facilities’ chief executive officers (CEOs) were obtained from the various provinces’ district health managers’ offices. The CEOs were contacted telephonically and electronically to be informed of their facilities’ inclusion in the audit. The CEOs were requested to inform their institutions’ medical managers of the pending research. Medical managers were identified as well suited to provide data on psychiatrists’ human resources and services at the institutions that they manage. The primary data were then obtained from key informant interviews with the medical managers of the health facilities on this list. Once participating medical managers’ informed consent was obtained, they were interviewed telephonically about the psychiatrist services and human resources at their facilities. Where medical managers were unavailable for telephonic interviews, an electronic format of the questionnaire was forwarded to them via e-mail. In the exceptional cases where facilities did not have medical managers, hospital CEOs or nursing service managers were requested to participate in the audit.

#### Instrument

The participants were interviewed using a structured questionnaire. The questionnaire was developed with the aim of gathering information about the MH human resources and services at participating institutions. The construction of this questionnaire was preceded by a literature review on the DOH’s strategic planning for the distribution of MH resources,^21^ and the WHO’s recommendations on situation analysis audit tools.^22^ The participants were asked the following questions relating to the psychiatrist services and human resources at their institutions:

- Do you have psychiatrists employed at your health facility?
- Do you have MOs dedicated to MH (MHMOs) care employed at your health facility? If so, how many do you have employed?
- Does your facility receive outreach psychiatrists services and at what frequency?
- How many sessions per week does your MHMOs have dedicated to MH?
- Are psychiatrists a part of your facility’s confirmed future staff establishment plan?

### Primary data analysis

The telephonic interviews were digitally recorded and transcribed followed by the transfer of these data onto the Fluidsurveys data management platform. The electronically completed questionnaires were uploaded directly onto the online data management platform. The data were collated, resulting in the creation of lists of psychiatrist human resources and services per province. The collated data were then transferred to Microsoft Excel’s Spreadsheet^®^ whereafter a descriptive, quantitative analysis of the psychiatric human resources and services were carried out using IBM’s SPSS 22^®^ package.

### Ethical consideration

Before the medical managers of the rural health facilities were interviewed, ethical approval for the study was obtained from the University of KwaZulu-Natal (UKZN) Biomedical Research Ethics Committee (BE416/13). Clearance to undertake research from each of the participating provinces’ research committees was then obtained. The nature of the study, confidential participation and voluntary withdrawal or refusal to participate were discussed with the potential participants and informed consent of all participants was obtained.

## Results

### Psychiatrist human resources and population sizes

The secondary data analysis indicates that by 2014, 762 psychiatrists were registered in SA,^23^ while it was not known exactly what percentage of this workforce is practising abroad.^24^ Approximately 40% of the country’s psychiatrists (302) are practising in the public health sector,^25^ responsible for over 44 million South Africans’ MHC.^2^ For the primary data, 160 out of 163 facilities representing the public rural health centres in SA participated in the situation analysis. A total of seven psychiatrists (2% of the total number of SA’s public sector psychiatrists) are employed in PRPHC health facilities that serves a population of 17 143 872 rural South Africans.^4,17^ Psychiatrists are represented at a rate of 0.03 per 100 000 population in SA’s public rural settings. A total of 99 (62%) PRPHC facilities receive no monthly psychiatric outreach by a psychiatrist. Table 1 shows the number of medical MH professionals working in the public sector’s rural health facilities per province and population size. With regard to outreach services, 31% (49) of facilities receive monthly visits by a psychiatrist, while 8% (12) received bimonthly visits (Table 2).

**TABLE 1:** Psychiatrist and MHMO human resources in PRPHC health facilities: province and population rates per 100 000 population.

Province	Rural population dependant on PRPHC health facilities		MHMOs		Psychiatrists	
		
	Population†	PRPHC facilities (*N*)	*N*	Rate	*N*	Rate
EC	3 333 810	31	10	0.30	0	0.00
FS	465 925	7	2	0.43	1	0.21
KZN	4 581 901	34	6	0.13	1	0.02
LIM	4 105 933	36	24	0.58	4	0.07
MPU	1 621 686	17	13	0.80	0	0.00
NC	173 248	10	0	0.00	0	0.00
NW	2 063 721	14	8	0.39	1	0.05
WC	797 648	11	0	0.00	0	0.00
**Total**	**17 143 872**	**160**	**63**	**0.37**	**7**	**0.03**

† , The rural population for each province making use of the public health sector was obtained by calculating the population of the health centres included in the audit, as well as that of their affiliated satellite clinics.

EC, Eastern Cape; FS, Free State; KZN, Kwazulu-Natal; LIM, Limpopo; MPU, Mpumalanga; NC, Northern Cape; NW, North West; PRPHC, public rural primary healthcare; WC, Western Cape; MHMO, mental health medical officer (not specialised in psychiatry, but dedicated to MH).

**TABLE 2:** The supplementary and outreach medical mental healthcare services at public rural primary healthcare institutions per province.

Variable	EC		FS		KZN		LIM		MPU		NC		NW		WC		Total	
								
	*N*	%	*N*	%	*N*	%	*N*	%	*N*	%	*N*	%	*N*	%	*N*	%	*N*	%
GMO weekly MHC sessions																		
None	30	96.8	7	100.0	26	83.4	34	94.4	17	100	10	100	13	92.9	11	100.0	147	91.9
One	0	0.0	0	0.0	0	0.0	2	5·6	0	0	0	0	1	7.1	0	0.0	3	1.9
Two	1	3.3	0	0.0	4	12.9	0	0.0	0	0	0	0	0	0.0	0	0.0	5	3.1
Three	0	0.0	0	0.0	4	12.9	0	0.0	0	0	0	0	0	0.0	0	0.0	4	2.5
Psychiatrists’ outreach sessions																		
None	27	87.1	3	42.9	16	47.1	9	25.0	17	100	10	100	13	92.9	4	36.4	99	61.9
Monthly	3	9.7	3	42.9	16	47.1	20	55.6	0	0	0	0	1	7.1	6	54.5	49	30.6
Bimonthly	1	3.3	1	14.3	2	5.9	7	19.4	0	0	0	0	0	0.0	1	9.1	12	7.5

EC, Eastern Cape; FS, Free State; KZN, Kwazulu-Natal; LIM, Limpopo; MPU, Mpumalanga; NC, Northern Cape; NW, North West; WC, Western Cape; MHC, mental healthcare; GMO, general medical officer.

### Supplementary medical human resources

Twenty-nine (18%) PRPHC facilities employ a total of 63 MHMOs (MOs dedicated to MHC – see Table 1). A further three facilities (2%) employ non-specialist general medical officers (GMOs) with a weekly dedicated session to MHC, five facilities (3%) employ GMOs with bi-weekly MHC sessions and four facilities (3%) have GMOs with three MHC sessions per week (Table 2).

### Future psychiatrist human resources

Nine PRPHC facilities (6%) indicated that psychiatrists are confirmed as employable specialists on their future staff establishment plans (Table 3). Over the last decade, 273 new psychiatrists were trained with an average output of 27 psychiatrists per year from 2005 to 2014 (Table 4).

**TABLE 3:** Psychiatrists confirmed on the future staff establishment of public rural primary healthcare health facilities per province.

Variable	EC		FS		KZN		LIM		MPU		NC		NW		WC		Total	
								
	*N*	%	*N*	%	*N*	%	*N*	%	*N*	%	*N*	%	*N*	%	*N*	%	*N*	%
Psychiatrist	0	0.0	0	0.0	0	0.0	6	16.7	2	11.8	0	0.0	1	7.1	0	0.0	9	5.6

EC, Eastern Cape; FS, Free State; KZN, Kwazulu-Natal; LIM, Limpopo; MPU, Mpumalanga; NC, Northern Cape; NW, North West; WC, Western Cape; MHC, mental healthcare; MH, mental health.

**TABLE 4:** Psychiatrists qualifying per year from 1995 to 2014.

Variable	Year										Total

	2005	2006	2007	2008	2009	2010	2011	2012	2013	2014	
Newly qualified psychiatrists	26	31	24	41	28	32	25	19	15	32	273

*Source*: Health Professions Council of South Africa^26^; Colleges of Medicine South Africa^27^

## Discussion

### Psychiatrist human resources

The official WHO-accepted South African psychiatrist to 100 000 population ratio is less than 0.5:100 000.^19,28^ Local and international studies suggest that this ratio is closer to 0.28:100 000.^7,29,30^ This ratio is significantly below the suggested standard for HMICs and when this ratio is compared to high-income countries (HICs) (15:100 000), where the number of psychiatrists is still deemed to be insufficient, it becomes apparent that SA’s population has desperately insufficient access to specialised prescribing MH professionals.^7^ By 2000, it was estimated that there was approximately one psychiatrist practising per 100 000 people in SA,^1^ making the supposed decline in per-capita psychiatrist numbers a concerning find.

A further worrisome factor is that according to the Human Resources for Health South Africa,^25^ less than 40% of the country’s psychiatrists are working in the public sector and this study suggests that the vast majority of these professionals are still centralised around urban areas, as the case was before SA became a democracy.^1,6^ Psychiatrist human resources are almost negligibly presented in SA’s PRPHC areas with a rate of 0.03 per 100 000 population. Four provinces (EC, MPU, NC and WC) do not have full-time psychiatrists employed in their public rural areas. By 2000, it was estimated that 4.7% of SA’s public service psychiatrists were practising exclusively in rural settings,^1^ and this study indicates that this percentage has decreased to around 2%. As suggested by other sources,^7^ this study corroborates that human resources for psychiatry in SA are at a critically underrepresented level, with the most dire access to this professional remaining in SA’s rural areas.^14,31^

### Efforts to relieve the medical prescriber crisis

From within the psychiatric profession, efforts have been made to increase access to their services in underserved areas by providing outreach services to rural PHC facilities. This study indicates that shortfalls remain with over 61% of public rural facilities receiving no monthly psychiatrist visits. In a further effort to alleviate psychiatric workforce shortages, the NDOH adopted the WHO-endorsed approach of task shifting,^12^ where the task of diagnosing mental illness and prescribing psychotropic medication was shifted to primary care MOs and MH nurses. While this approach has been found to be effective in rural communities,^13,32^ this study indicates that there is a shortage of MOs dedicated to MH to whom this task can be shifted. Only 19% of rural public facilities have MOs dedicated to MHC, which is disconcerting, especially when considering the findings of a recent study that MH nurses are also underrepresented, being employed at only 42% at these facilities.^33^ This suggests that a substantial amount of mental healthcare users visiting rural public facilities do not receive care (including diagnosis and psychopharmacological intervention) from dedicated MH professionals. Even with the concerted efforts made to improve access to psychiatric care, critical human resource shortages remain in SA’s rural public health facilities.

### Psychiatrist output and training

Psychiatrists are medical specialists who require at least 13 years of rigorous training in SA to qualify as independent practitioners. The duration and intensity of their training contribute to the output of new psychiatrists trained over the last decade averaging at a modest 27 per year.^27^

Even though psychiatry has historically been a popular speciality choice in SA,^1^ the supply of psychiatrists is not keeping up with the population’s demands. Budgetary constraints, limited training spaces available for psychiatric registrars and an NDOH focus on medical rather than MH interventions are among the most discussed contributing factors.^6,7,34^ The last decade has seen no increase in the annual average number of psychiatrists trained, with the last five years indicating a slight decline.^27^ With an average output of 27 psychiatrists per year over the previous decade,^27^ the rate of psychiatrists per population is likely to decrease when compared to the population growth estimates.^17^ It would therefore be unrealistic and impractical to rely solely on an increase of psychiatrists to bridge the MH treatment gap in SA’s rural areas.

### Recommendations

This study indicates that even though the need for upscaling psychiatric human resources in rural areas is vital, it will be a lengthy process that will have to be supported by a variety of MH professionals. It also suggests that the current efforts to alleviate this workforce crisis are falling short of the target of providing an inclusive MH service for all South Africans set forth by the NDOH at the dawn of SA’s democracy.^1^ With the dire shortages of psychiatrists in rural areas and the lack of MOs and MH nurses^15^ to whom the task of diagnosing mental illness and prescribing psychotropic treatment can be shifted, current task shifting approaches need to be reconsidered and innovated. Recently, the NDOH and the HPCSA considered more creative solutions to alleviate workforce shortages. Limited prescriptive authority has been granted to clinical associates, a mid-level health practitioner, working in PHC areas, to include the limited use of medications for MH conditions.^25^ This can be seen as a type of collaborative prescribing where if medication is classified as scheduled to be potentially hazardous or not on the essential drugs list, a MO is required to countersign the script. It is recommended that in the light of the severity of the MH workforce shortages, further, similar innovative approaches to task shifting be considered: other HMICs and HICs have considered including more cadres of healthcare professionals to whom traditional medical tasks could be shifted at the PHC level. Pharmacists and clinical psychologists, specifically trained in clinical assessment and psychopharmacology, have been employed internationally to assist with prescriber workforce shortages and found to be safe, effective and practical.^36,37^ In SA’s rural public settings, clinical psychologists are employed in at a rate 11 times the rate of psychiatrists.^15,38^ This cadre shares psychiatrists’ primary treatment goal of alleviating distress in mental illness and could possibly help to alleviate prescriber shortages if appropriately trained. Even though this cadre is trained in MH assessment, diagnoses, the basic neurosciences and psychopharmacology, they traditionally focus on psychosocial interventions. Specialised, advanced psychopharmacologically trained clinical psychologists would seem an appropriate cadre to whom the task of prescribing psychotropic medications at a PHC level could be shifted, but to ensure patient safety^1,39^ it is proposed that psychiatrists and family physicians are involved in the construction of training and supervision programmes for all cadres of non-medical prescribers.

### Limitations to the study

While this audit attempted to describe the psychiatric human resources and services in all of SA’s rural public settings, it has to be considered that, with this audit’s duration of two years and the dated government document^20^ used as the basis of facility inclusion, the list may not be exhaustive. The facilities’ inclusion was primarily based on the only official government document available for public perusal (it was just less than a decade old at the start of the audit). Even though the authors attempted maximum inclusion by involving the provincial DOHs in the process of constructing updated lists of rural facilities, some may have been omitted. Notwithstanding, with a participation rate of 98% of the facilities included in this audit, the results can be considered to illustrate a representative overview of SA’s rural medical prescribers’ human resources and services. Generalist MOs employed at PRPHC facilities, expected to assist in providing primary MHC, were not included in this audit as only dedicated MH care professionals were considered in this study. Furthermore, the calculation of the different province’s rural population making use of the public health system may be underrepresented, as SA’s rural population, being its poorest population, has a smaller percentage of the population making use of private medical care than the SA norms.^17,25^ This conservative rural population calculation may then indicate that the treatment gap between human resources for psychiatry and mental illness’ burden of disease is actually greater than that proposed in this paper.

## Conclusion

The psychiatrist workforce is severely underrepresented in SA’s rural PHC public areas. Efforts to integrate MHC into PHC have been falling short of its targets. The number of psychiatrists working exclusively in rural areas has decreased over the last decade while the annual output of psychiatrists has not been able to keep up with the population’s demands. Despite strategies to alleviate the psychiatric workforce shortages through task shifting, substantial treatment gaps remain within psychiatric human resources and services. The majority of rural public health facilities are failing to employ prescribing health practitioners dedicated to treating mental illness. Recommendations are made to re-evaluate the current task shifting approach by considering more non-medically trained professionals – who could undergo specialised training in the neurosciences and psychopharmacology – to fill the psychiatric workforce need in public, rural health facilities. It is imperative that psychiatrists and family physicians are involved in the development of training programmes and supervision of prospective non-medical prescribers.
